# Supplementary material for: Protection of Mild Steel by Waterborne Epoxy Coatings Incorporation of Polypyrrole Nanowires/Graphene Nanocomposites
Source: Polymers (Basel). 2019 Dec 3;11(12):1998. doi: 10.3390/polym11121998 (PMC6960955; doi:10.3390/polym11121998)
Supplement: Supplementary file 1 [file polymers-11-01998-s001.pdf]

Supplementary material:

Polypyrrole nanowires and epoxy resin coatings (15 mm × 10 mm × 2mm) with different proportions were prepared and soaked in deionized water. The water absorption rate was obtained by weighing the increased weight (m_t) at a certain time interval. The water absorption rate (A_t) was calculated according to the following Equation 4:

$$A_t = \frac{m_t - m_0}{m_0} * 100\%$$

where m_0 is the mass before immersion.

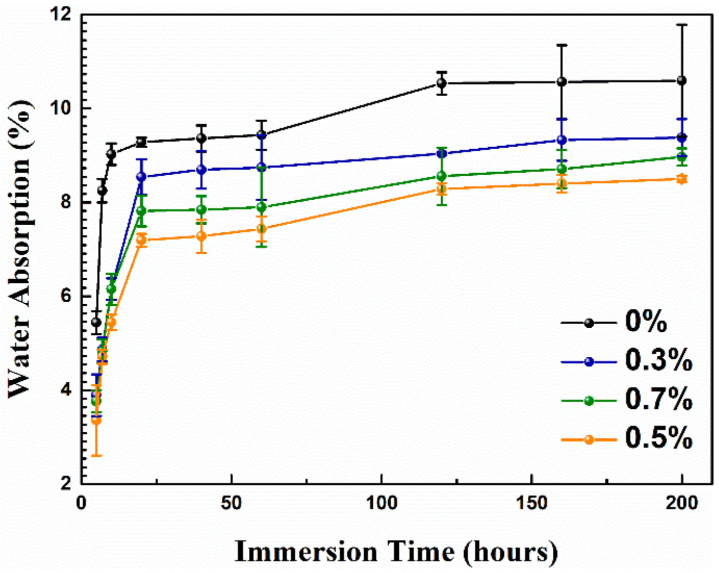

Figure S1. Water absorption of epoxy/polypyrrole nanowires coatings filled with 0%, 0.3%, 0.5% and 0.7% polypyrrole nanowires.

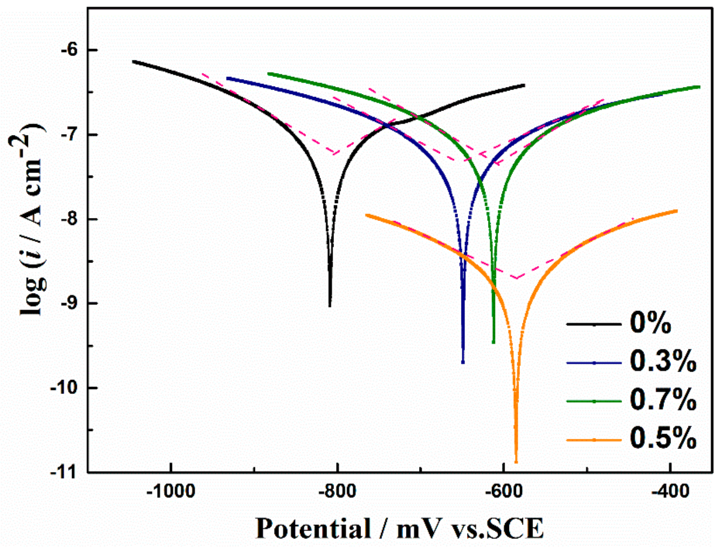

Figure S2. Tafel curves of mild steel with coatings of epoxy/polypyrrole nanowires coatings filled with 0%, 0.3%, 0.5% and 0.7% polypyrrole nanowires in 3.5% sodium chloride solution after 20 days.

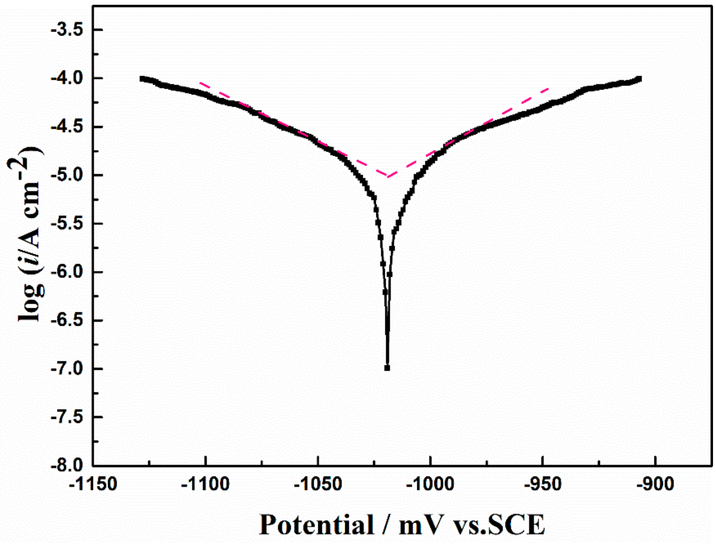

Figure S3. Tafel curve of the bare mild steel electrode.

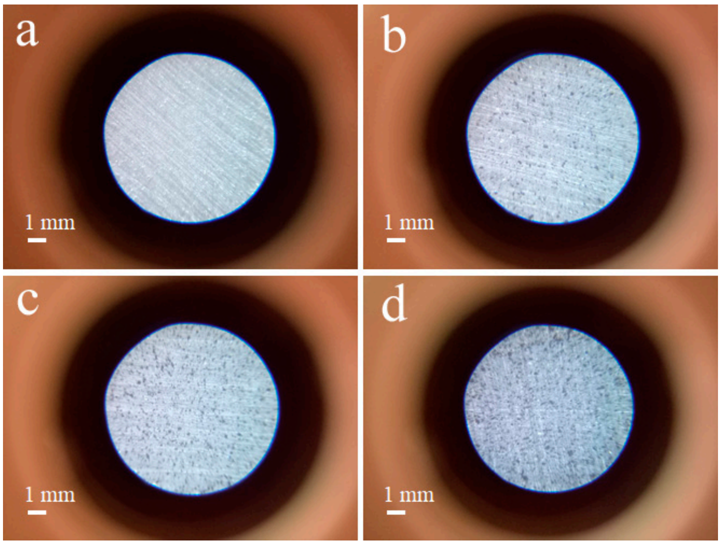

Figure S4. Optical microscopic images of epoxy/polypyrrole nanowires coatings filled with 0% (a), 0.3% (b), 0.5% (c) and 0.7% (d) polypyrrole nanowires.
